# Will E-Monitoring of Policy and Program Implementation Stifle or Enhance Practice? How Would We Know?

**DOI:** 10.3389/fpubh.2018.00243

**Published:** 2018-09-11

**Authors:** Kathleen P. Conte, Penelope Hawe

**Affiliations:** ^1^The Australian Prevention Partnership Centre, Sydney, NSW, Australia; ^2^Menzies Centre for Health Policy, School of Public Health, Faculty of Medicine and Health, University of Sydney, Sydney, NSW, Australia; ^3^O'Brien Institute of Public Health, University of Calgary, Calgary, AB, Canada

**Keywords:** implementation, health information technology, health promotion, quality improvement, accountability, innovation, program monitoring

## Abstract

Electronic or digital monitoring systems could promote the visibility of health promotion and disease prevention programs by providing new tools to support the collection, analysis, and reporting of data. In clinical settings however, the benefits of e-monitoring of service delivery remain contested. While there are some examples of e-monitoring systems improving patient outcomes, the smooth introduction into clinical practice has not occurred. Expected efficiencies have not been realized. The restructuring of team work has been problematic. Most particularly, knowledge from research has not advanced sufficiently because the meaning of e-monitoring has not been well theorized in the first place. As enthusiasm for e-monitoring in health promotion grows, it behooves us to ensure that health promotion practice learns from these insights. We outline the history of program monitoring in health promotion and the development of large-scale e-monitoring systems to track policy and program delivery. We interrogate how these technologies can be understood, noticing how they inevitably elevate some parts of practice over others. We suggest that progress in e-monitoring research and development could benefit from the insights and methods of improvement science (the science that underpins how practitioners attempt to solve problems and promote quality) as conceptually distinct from implementation science (the science of getting particular evidence-based programs into practice). To fully appreciate whether e-monitoring of program implementation will act as an aid or barrier to health promotion practice we canvass a wide range of theoretical perspectives. We illustrate how different theories draw attention to different aspects of the role of e-monitoring, and its impact on practice.

## Introduction

*The air-conditioning unit in the portable office shudders, then dies. It's 6pm and 40*°*C. The health promotion practitioner groans but doesn't look up from her computer. She's rushing to record today's work before the end-of-month deadline for her supervisor, located 200kms away. While the documentation system loads, she shuffles in her bag, through health pamphlets and educational aids, to locate the participant satisfaction evaluations from today's health fair. Clicking through drop-down lists across multiple screens, she inputs the scores. She then tallies and enters the number of people who registered for her nutrition newsletter. Logging out, she creates a reminder in her phone to follow-up with the cancer council about next week's smoking cessation program. Before leaving she handwrites a sticky-note for her colleague: “Low turnout–Hot! Probably won't meet our targets this month. PS. The aircon is dead – again* :(”^1^

## Background

The growing sophistication of digital technologies has generated wide interest among the public health practice sector for monitoring the delivery of health-promoting services and policies. Governments and non-government organizations have invested in digital technologies in the form of digital monitoring systems to track and oversee the quality and delivery of health promotion services—primarily evidence-based policies and programs (1, 2). Digital monitoring systems are able to collect, record, analyse and communicate real-time data about program and policy implementation, for multiple stakeholders located across vast distances (3).

Despite the promise of these systems to improve communication and practice, there are growing examples of failed digital monitoring systems from both clinical and health promotion settings (2, 4–6). Digital monitoring technologies in the clinical sector, e.g., electronic patient records, promised to improve service delivery, lower costs, and improve health, however, this promise has not been fully realized. Some systems have failed to produce, or even worsened, health outcomes (4, 5). Others have floundered when faced with the complex day-to-day intricacies of practice (2), or failed to maintain relevance in response to program shifts (6). This is despite many billions of dollars of investment in the design and roll-out of digital monitoring systems (7).

Nevertheless, the use of e-monitoring systems to oversee the implementation and delivery of health promotion programs is growing. The opening scenario of this paper will be common place to many readers who are already using such e-monitoring systems, or who may have developed their own digital systems for tracking the delivery and reach of programs and activities. These technologies can be classified as “health informatics,” a type of digital health technology to which electronic patient records are also a member (8) (see Table 1 for a glossary of terms used throughout this article). While electronic patient records monitor the delivery of healthcare to individuals, in the context of health promotion, electronic monitoring systems (hereafter referred to as “e-monitoring”) are used to track the delivery of preventive health programs, services, and activities to populations and settings, for example, communities, schools, or work places. However, information about the design, use, and impact of these systems has received little attention in the academic literature. As enthusiasm for e-monitoring systems grows, it behooves the field of health promotion to consider the phenomenological and epistemological questions about the use of digital technologies that have arisen in other sectors (8). For example, how do e-monitoring systems change the actions and relationships of practitioners? How are concepts of population health and health promotion challenged or reinforced through the design of e-monitoring systems and the data they capture? What are the implications for knowledge and power dynamics between communities, practitioners, and policy-makers? The purpose of this paper is to consider how e-monitoring technologies might impact the field of health promotion, and to suggest areas for future research. We do so by (1) providing examples of how key e-monitoring systems have developed and are currently used in health promotion practice, (2) reviewing the role of monitoring in health promotion, (3) examining whether e-monitoring systems might facilitate or hinder the act of monitoring, and (4) anticipating and articulating different theoretical lenses we may use to detect the intended and unintended impact of e-monitoring.

**Table 1:** Glossary of terms.

**Term**	**Definition**
Electronic Monitoring (e-monitoring)	The use of electronic computer software or systems to conduct monitoring activities
Continuous Quality Improvement^a^	“Continuous and ongoing effort to achieve measurable improvements in the efficiency, effectiveness, performance, accountability, outcomes, and other indicators of quality in services or processes which achieve and improve health of the community”(9) A comprehensive management philosophy that focuses on continuous improvement by applying scientific method to gain knowledge and control over variation in work processes (10)
Digital Health Technologies	Electronic devices used to track deliver, track, manage, and collect information used in the delivery of health services, or in endeavors to promote wellness. Used as an overarching term for multiple types of technologies that perform specific functions, e.g., electronic patient records, web-based program management and data collection systems (8)
Health Informatics	The use of digital technologies to collect, analyze and communicate health information and data (8)
Implementation Monitoring	The oversight of the delivery of interventions. Definitions vary, and may include some or all of the following: the delivery of components, the (number and type of) people reached, the intensity or “dose” of effort being applied, the circumstances surrounding delivery and the key milestones achieved
Implementation Science	“The scientific study of methods to promote the systematic uptake of research findings and other evidence-based practices into routine practice, and, hence, to improve the quality and effectiveness of health services” (11)
Improvement Science^b^	The systematic examination of the methods and factors that work best to facilitate quality improvement (12)
Monitoring	“A continuing function that aims primarily to provide the management and main stakeholders of an ongoing intervention with early indications of progress, or lack thereof, in the achievement of results” (13)
Quality Assurance/Quality Control	Systematic monitoring and evaluation of performance of an organization or its program to ensure that standards of quality are being met (14)

a Two definitions are given to recognize the historic concern with (unwarranted) variation between different settings.

b *Note that this does not have to specifically include uptake of any particular evidence-based program. Improvement science has a focus on the systematic examination and interpretation of actions to improve quality and effectiveness, whereas some traditional definitions of quality control and quality improvement may be action-focused only (with less emphasis on using and adding to the science of the action)*.

## Examples of E-Monitoring systems in health promotion practice

The earliest application of e-monitoring systems to monitor the delivery of health promotion programs and activities used generic software applications—e.g., word processors, spreadsheets and database software. Significant resources were spent in developing bespoke templates and protocols using these applications to collect monitoring data across sites and to train users (15). In Table 2, we provide examples of types of e-monitoring systems that are currently in use in the health promotion context.

**Table 2:** Examples of software systems in use to support e-monitoring of health promotion implementation.

**Software**	**Publication/web resource**	Description^*^	Evidence of use in practice^**^
**COMMERCIAL SYSTEMS, CUSTOMIZABLE FOR MONITORING PURPOSES**			
Generic Application Software. Examples include Microsoft Office, Apple Apps, Google Drive	Microsoft Office: https://products.office.com/en-au/products Apple Apps: https://support.apple.com/apps Google Drive: https://www.google.com/sheets/about/	Free or fee-for-service software that provides basic but customizable computing functions including word processing, spreadsheets, databases, PowerPoint, and web design.	Used to collect store, and manage data. The development of templates in these applications have been used to facilitate large-scale data collection and reporting. For examples of use, see Fernald et al. (15).
Survey Software	https://www.Qualtrics.com https://www.Surveymonkey.com	Customizable survey platforms that allow users to create bespoke surveys, collect and analyse data, and create reports.	Used to collect online survey data. For examples of use, see Brownson et al. (16).
Project Management Software	https://www.Basecamp.com	Web-based, project management tool that provides a central platform for project partners or staff to communicate, plan, track progress, and store files.	Used by Bors et al. (6) to coordinate the development of the Health Kids, Healthy Communities monitoring system.
**BESPOKE MONITORING SYSTEMS**			
Healthy Kids, Healthy Communities Community Dashboard	Bors et al. (6)	Web-based documentation and networking system designed to track progress and facilitate communication between and among administrators and recipients of the Robert Wood Johnson Foundation's Healthy Kids, Healthy Communities grant scheme.	Project-specific monitoring system that was discontinued after the end of the grant program.
Population Health Information Management System	Farrell et al. (1)	Web-based documentation system that records adoption of key performance indicators of physical activity and nutrition policies by day care centers and primary schools. Local health districts use the system to plan, tailor and monitor local service delivery, and to report their progress to the Ministry of Health.	Developed by the New South Wales, Australia Ministry of Health, this system is used by local health districts to document and report progress in achieving the key performance indicators.
**SOFTWARE-AS-SERVICE MONITORING SYSTEMS**^a^			
EvaluationWeb	http://www.lutherconsulting.com/evaluationweb.html	Fee-for-service, customizable online data collection and reporting service.	Used by several US states' health departments to collect HIV/AIDS prevention and treatment program data and to coordinate reporting requirements for the Centers for Disease Control and Prevention (CDC).
Compass by QTAC, NY	https://compass.qtacny.org/	Fee-for-service, online database that collects and stores data about evidence-based chronic disease self-management workshops. Provides a list of current self-management programs, allows instructors to download required program forms and input participant data, and allows medical providers to refer patients to programs and to receive updates. Aggregates and reports data directly to CDC to fulfill reporting requirements for their grantees.	Currently in use by the state of New York health department and by the Oregon Health Authority to coordinate data collection to fulfill reporting requirements to the CDC.
Quality Improvement Program Planning System (QIPPS)	https://www.infoxchange.org/au/products-and-services/project-management	Fee-for-service, online project planning and evaluation database designed specifically for health promotion project planning and management activities.	At least two Australian state governments have used QIPPS to track and report on case studies of community-based health promotion projects, see Round et al. (17) & Northern Territory Government of Australia (18).
DevResults	https://www.Devresults.com	Software-as-service program for monitoring and evaluation and project management. Collects raw data and tracks progress by location against selected indicators, includes project management functions including budget tracking, task assignment, and document storage.	Used by large international development organizations for tracking international aid programs.
**OPEN-SOURCE MONITORING SYSTEMS**^*b*^			
DHIS 1 & 2	https://www.dhis2.org/inaction	Free, open-source, software to collect, validate, analyse and present aggregate and patient-based statistical data. Highly customizable to aggregate and track site-level data in addition to patient-level data. Mobile capability available for patients and providers to manage eHealth records.	Adopted by Kenya, Tanzania, Uganda, Rwanda, Ghana, Liberia, and Bangladesh, as their primary national health information system. Used in >40 countries.

* *Drawn from information from the companies' website in the public domain, or from literature where available*.

** *Examples drawn from the literature where possible, and from practice-based knowledge and experience of the authors*.

a *Software-as-service is a model in which software is centrally-hosted and supported by the vendor, and license fees are paid via a subscription*.

b *Open-Source refers to computer software where the source code is freely accessible and available for use by the general public. It can be changed and used by anyone, for any purpose, without a license. To our knowledge, there are no examples of open-source software for e-monitoring of health promotion. The DHIS is presented as an example of open-source software used in clinical settings. Because it is open-source it is highly modifiable and could be adapted to the health promotion context*.

Overtime, there was a push to streamline data collection and reporting into online data-management systems funded in part by health promotion infrastructure, e.g., governments and large organizations (1, 2). Commercial software companies began to offer adaptable data management systems capable of data analysis, project management, and real-time reporting functions. The advent of open-source software gave rise to free software systems that can be customized by local computer scientists to fit local needs for health monitoring. Both commercial and open-source systems are regularly used by health promotion researchers and practitioners to collect and present data about reach and facilitate workflow and collaboration between stakeholders (6, 16).

Despite the increased sophistication of software tools and their use in health promotion, few are sufficiently described in the academic literature. This is particularly true of bespoke systems that are developed for internal use. Often, software is created, used and abandoned or morphed into new systems without a record of the purpose it served, the lessons learned from its use, or the reasons for its failure (19). For example, the Program Evaluation and Monitoring System (PEMS) developed by the Centers for Disease Control and Prevention was meant to facilitate monitoring and assessment of the national HIV/AIDS prevention program in the United States (2). PEMs was meant to standardize reporting about HIV/AIDS counseling interventions and client details (e.g., risk behaviors and service use) delivered by local agencies across the USA. The burden of data entry, however, met with strong resistance from community organizations (20), and despite the expense dedicated to its development, PEMS never fully launched. The reasons for this, however, are not described in the literature.

Information about the development, use, success and failure of e-monitoring systems is needed to guide practitioners who wish to develop or purchase software to facilitate monitoring. Lyon et al. (21), recognized there was a gap between commercially-developed health software, and academic research on the topic. They developed a methodology for evaluating “measurement feedback systems,” or digital systems that routinely monitor outcomes in the health service sector. This methodology seeks to bridge commercial computer industries and academics by providing a tool with which researchers can identify and evaluate the capabilities of different computer monitoring systems for use in monitoring clinical outcomes. A similar but adapted methodology is needed in health promotion.

One example of e-monitoring in health promotion is illustrated by Brennan et al. (22) who developed a web-based computer system to monitor the activities of 49 funded community partnerships across the United States. They developed a typology of implementation that weighted the dose of intervention delivery to reflect the scale of reach, quality of implementation, and the potential impact of interventions undertaken across the communities. The utility of the e-monitoring system among users, however, was not as beneficial as it was to researchers, and it was disbanded after the end of the grant program (6). This highlights one of the key problems in the design of e-monitoring systems for health promotion: what role is e-monitoring expected to play in practice, and whose needs does it meet? To answer, we must consider what monitoring is, and what it is intended to do.

## The role of program monitoring in health promotion

Throughout the history of health promotion, monitoring activities and their outcomes has been part of practitioners' day-to-day practice. In some cases, years before clinicians were being asked to engage in evidence-based practice (23), health promotion practitioners were doing needs-based planning and designing logic-models for interventions (24). They were designing evaluations of process (e.g., reach, implementation, satisfaction and quality) (24, 25), assessing short term effects (impact evaluation) and achievement of long term goals (outcome evaluation). The ability of practitioners to plan, track and adjust their approach to practice was enshrined as a professional competency (26–28). Programs were monitored, targets of change (i.e., risk factors) were monitored, and even some of the behind-the-scenes work of practitioners in capacity building and the creation of inter-organizational collaborations came to be measured, though not as part of routine surveillance (29). As outcome evaluations of programs accumulated, meta-syntheses produced recommendations for best practice (30) as well as impetus to design monitoring systems to ensure effective programs were being implemented with fidelity, and reaching their intended audience (31).

The emphasis on monitoring fidelity, however, highlights a perennial tension that has existed throughout health promotions' history between “top-down” vs. “bottom-up” approaches to best practice (32). Top-down approaches, led by policy makers, identify best practice through research and then devise ways to diffuse, facilitate and incentivize the faithful delivery of best practice programs by practitioners. Bottom-up approaches assume that the best approaches to achieving health gains are discovered through the trial-and-error learning methods of practice now enshrined in models like the “plan-do-study-act” cycles (33). While many scholars saw the inevitability and even the benefit of this tension (34), they also foresaw that increased monitoring could exaggerate it. This would happen when one side (usually the top-down) developed stronger monitoring capacity than the other, and prioritized measuring phenomena seen as antithetical to, or not sufficiently representative of, what local practice might wish to achieve (35, 36).

Ottoson (37) has argued that top-down approaches to health promotion are heavily influenced by knowledge utilization theory and particular types of transfer theories which use fidelity of form as the criterion for success. In other words, with top-down approaches (and monitoring systems designed to support this), ideally the program or policy is unchanged by context. By contrast a bottom-up approach takes a more political and social understanding of change, where adaptation to context is a driver of success (37). Hence monitoring systems would have to accommodate (indeed encourage) the recording of diversity in practice. Expressed in the terminology of complexity, with bottom-up approaches, the agents in the system are viewed as problem solvers with power and decision making abilities that are seen to appropriately eclipse pre-determined or standardized solutions. By contrast, top-down approaches see the health promotion “system” as complicated -not complex- and its various parts expected to be faithfully reproduced.

In the real world, there are probably no such absolutes. But the insights are helpful for navigating current debates and distinctions between implementation science and improvement science (38, 39). Implementation is the science of getting particular evidence-based programs into practice (11); it tends to focus on the faithful replication of core components of programs (38). By contrast, improvement is the science that underpins how practitioners attempt to solve problems and promote quality (12). Improvement science is about sensitizing practitioners to discrepancies between “what is” and “what should be” and building strategies of action to meet desired goals (39). “What should be” can include more faithful adoption of evidence-based programs, but it can also extend to other activities, such as the restructuring of organizational culture to create more opportunities to reflect on performance (40).

The current day distinction between implementation science and improvement science is reminiscent of earlier-day distinction made by Stephenson and Weil (41) between systems of practice which rely on the replication of “dependent capability” (people working on familiar problems in familiar contexts) in contrast to practice systems which foster “independent capability” (ability to deal with unfamiliar problems in unfamiliar contexts). The former fits with implementation science. The latter aligns with improvement science. Add to this now the real-time ability of e-monitoring systems to privilege one type of practice process over the other, with fast collating monitoring systems that amplify differences in approach. Health promotion is thus left to ponder the question of what type of knowledge generation do we wish to advance and therefore, capture and enshrine in the design of subsequent e-monitoring systems? One narrowed to measuring the transfer and impact of particular programs only? Or one that recognizes that, at the local level, there may be a diversity of actions and innovations, some of which worth capturing and developing further?

## How might E-Monitoring systems enhance or impede the purpose of “monitoring”?

A clear advantage of e-monitoring systems is that they potentially offer health promotion increased visibility at high bureaucratic levels, in a health sector currently dominated by clinical services. E-monitoring systems may bestow more authority to health promotion (1). Their use could signal a step out of the margin and into the mainstream. More than that, the systems provide high-level decision makers new information that potentially shines a favorable light on health promotion. Viewed alongside statistics on surgical waiting lists, or the growing size of the pharmaceutical costs, e-monitoring systems can tabulate the number of schools tackling obesity or the number of childcare centers with active play policies.

However, the design of an e-monitoring system will also determine what activities and practices get recognized. The competing priorities of different stakeholders raises potential concerns. Practitioners likely need different information to inform their immediate work (e.g., practical information about managing a task) than their managers at a government level (e.g., information about reach and target achievement). For example, in the opening scenario of this paper some of the most important pieces of information were written by hand on a sticky note—not entered into the e-monitoring system. The inherent complexity of health promotion in practice (42) requires monitoring systems that maintain confidence at high bureaucratic levels, while simultaneously enabling candid exchange of information at the practice-level. Indeed, practice-level information, e.g. uncertainties encountered, relationships formed and lost, frustrations, time wasted, could be (mistakenly) interpreted as indicative of goal slippage.

There is also a strong literature in capacity building for health promotion which indicates the importance of investing in generic activities that lead to multiple benefits (43). This means that the time a health promotion practitioner invests in building relationships with local organizations to deliver on nutrition targets could simultaneously be drawn-upon to address problems regarding tobacco or social inclusion issues. It follows that e-monitoring systems designed to entrench the tracking of high-priority health problems may ultimately crowd and compete with each other in a space where practitioners invest their time in ways that cannot be reliably attributed to any particular silo anyway (43).

The risk then is that e-monitoring systems meant only to track spending or count deliverables will likely fail to detect and fail to recognize key health promotion activities. In doing so, e-monitoring systems could not only reduce the value of health promotion work to a series of pre-defined, quantifiable measures, but also shift practice toward achieving these measures and away from continuous quality improvement and innovation. Maycock and Hall (36) caution against the development of a “tick-the-box mentality” in performance monitoring, with practitioners being “locked into and rewarded for current behavior patterns rather than creatively looking for alternative methods of improving outcomes” (p. 60). This statement marks the difference between passive performance monitoring and active processes of continuous quality improvement (CQI). In CQI, practitioners use the data reflexively to interrogate their work and innovate, hence reshaping the nature of their practice. Program delivery tracking and target assessment can still occur, but hopefully in a way that will not counteract more broadly focused CQI processes.

It is not a limitation of e-monitoring systems that they preference the collection and reporting of quantifiable, “tick box” indicators. It is simply a characteristic, and one that continues to change as technology increasingly enables the collation and visual representation of data. But no matter how well-designed, an e-monitoring system is simply a tool that facilitates the collection, management, and communication of data. Like any other tool, its optimal utility is achieved only when its design is appropriately matched to both task and user, and its function is clear. The design of the various e-monitoring systems described in Table 1 likely reflect their original purposes; the use of these systems will naturally pull practice toward some activities more than others. The actual application of these systems will differ in practice, depending on how and for what the user uses them. So while the design of e-monitoring is critical, of primary importance is articulating the purpose the act of e-monitoring is intended to perform.

## How theory answers the question: will E-Monitoring stifle or enhance practice?

Theory underlies “all human endeavors,” including endeavors of quality improvement (44). Yet often, the theories that underlie improvement efforts are not explicitly stated and go unrecognized. Articulating the theory that underlies an improvement effort enables us to uncover contradictory assumptions or incoherent logic in a program of action (44). Therefore, it is necessary to explicitly state the *how* e-monitoring is intended to facilitate improvement.

Previous scholars have illustrated the importance of making explicit the theoretical paradigm that underpin research processes used to investigate the act of e-monitoring. For example, work by Greenhalgh and colleagues illustrates how different types of knowledge about EPRs were generated via the application of different research paradigms (see Table 3) (45). This field is of interest to health promotion as the EPR can be thought of as the clinical analog of a health promotion program record. It records descriptive data about the patient as well as what advice, services and procedures have been dispensed to the patient. During a landmark synthesis on EPR literature, Greenhalgh and colleagues classified previous studies according to nine meta-narratives, each stemming from different historical and philosophical roots. The results reflected a wide range in how EPR research had been conceptualized, conducted, and ultimately, informed the current direction of EPR use and subsequent research. Of particular interest is their idea that the EPR studies reflected a tension between two framings (45). In one framing, technology is considered to have inherent properties that will perform certain tasks and improve processes and outcomes in more or less predictable ways across different settings. In the other, technology has a social meaning derived from the context of how it is used in practice. The important implication is that the ability to understand the range of impacts that e-monitoring can have on practice will depend on the research paradigm(s) used to detect it.

**Table 3:** Different philosophical research traditions observed to underpin electronic patient record research^*^.

**Research tradition**	**Ontological and epistemological assumptions**
Positivist	An external reality can be known and objectively measured
Interpretivist	“Reality” is inevitably understood/represented through the researcher's values, experience and identity
Critical	Research/facts/knowledge typically privilege the viewpoint of those in power. Critical perspectives challenge this
Recursive	“Reality” is generated within social structures that are reciprocally and recurrently reproduced by people's actions

* *Following Greenhalgh et al. (45), taking ontology to be assumptions about the nature of reality and epistemology to be how that reality might be known*.

Ultimately, it is through the use of theory that we may answer the question posited in our title: How will we know if e-monitoring of policy and program implementation stifles or enhances practice? In short, the answer depends on how we theorize what practice is and how a particular e-monitoring system's logic-of-action then fits with practice. In other words, we must articulate the mechanisms by which e-monitoring is intended to bring about change and improve practice so that assumptions can be verified, and the relationship between the act of e-monitoring and its intended outcome(s) can be tested.

In Table 4 we illustrate some key theories we think are relevant for determining what we might glean about the act of e-monitoring. Note these theories concern the act of e-monitoring itself, not the programs or policies being monitored. Each theory challenges what the act of e-monitoring means, and in what ways it may impact practice. For example, Worldview Theory would invite consideration of what the e-monitoring system asks a practitioner to do, and whether this clashes with core practice values. Patterson and colleagues used Worldview theory to show how no-smoking policies in hospitals are stymied by the security staff who were meant to enforce them when these staff were unwilling to go against a higher order value of protecting the “downtime” (and private smoking behaviors) of nurses and doctors whom they held with the highest regard (62). In the health promotion context, an e-monitoring system which embeds siloed practices aimed at particular “risk factors” might not be well used if it clashes with more traditional “bottom up” practice values.

**Table 4:** Key theories for considering what the act of e-monitoring means in practice.

**Theory**	**Some Key Citations**	**Focus**	**Relevance to the E-monitoring of Health Promotion Practice**	**Example Research Questions**
Fit Between Individuals, Task and Technology (FITT) *Behavioral science theory*	Ammenwerth et al. (46)	Suggests that a new technology leads to positive changes only if the attributes of the user group, the characteristics of the implemented technology, and the associated tasks match each other.	Features in Davidoff et al. (44) as a case example of the use of theory within one part of a Plan-Do-Study-Act cycle of a quality improvement project. Led to the achievement of a national target in health care treatment quality.	What are the characteristics of high users of the e-monitoring technology?
Institutional Theory *Political theory*	Scott et al. (47)	Concerned with how the most deep and resilient aspects of social structures (e.g. schemas, rules, behaviors routines etc.) are created and maintained.	Of interest because in spite of the dubious effectiveness of electronic records in the health system, their transfer into health promotion will likely increase the legitimacy and authority of health promotion.	What is the highest level of authority in the state health department at which data from e-monitoring of health promotion is used? What are the ripple effects of this?
Practice theory *Sociological theory*	Feldman and Orlikowski (48)	Examines the “constitutive” role of practices in producing organizational reality. Social life is the product of ongoing recurrent actions.	Implication is that health promotion practice will be shaped and ‘recreated' by digital implementation monitoring.	How does e-monitoring fit with existing practice? How will practice outside of the digital fields been maintained (or not)?
Structuration theory *Sociological theory*	Giddens (49)	Considers that social structures (relationships, traditions, moral codes etc.) are the product of human agency (thoughts, decision-making, power) and vice versa. Larger social structures are the product of the repetitions of actions by individuals at micro levels.	Shares similarity with other theories, but often quoted when drawing attention to individual agency.	How is individual practitioner agency impacted by e-monitoring? What is the consequence of any shift in agency?
Normalization process theory (NPT) *Sociological theory*	May (50); May et al. (51);	How an organizational practice, classification, technique or artifact gets embedded into everyday life. Articulates associated mechanisms including coherence (sense-making), cognitive participation (relationship work); collective action (work to enact the practice) and reflexive monitoring (appraisal of the effects).	Considers implementation as a social process of collective action. NPT has been used extensively in understanding uptake of innovations in clinical practice and to explain factors that promote or inhibit the implementation of e-health systems. (52)	What is the “talk” that accompanies e-monitoring in health promotion? How does it enact and embed the practice of monitoring?
Actor network theory *Sociological theory*	Callon (53); Bilodeau and Potvin (54);	Considers how relationships between people are forged and continuously reshaped by the use of non-animate entities (like a technology).	Invites a focus on the way professional practice networks and intersectoral partnerships respond to the introduction of e-monitoring.	How has e-monitoring expanded or concentrated/centralized networks of practice? Have existing network structures influenced the adoption of e-monitoring?
Activity settings theory *Community psychology theory*	O'Donnell et al. (55)	Similar to structuration theory and practice theory, examines the everyday settings of life where the dynamic interaction of people and things produces regularized “scripts” or behaviors/practices/expectations.	Provides a systematic architecture for examining the properties of an activity setting. Unlike structuration theory, an advantage of activity settings theory for health promotion is that it provides guidance about design of ecological interventions (interventions that focus on the properties of the context, not the people in them). This architecture also provides a scheme to analyse how digital implementation monitoring impacts on some key features of the setting e.g., roles, resources, and the symbols, and time.	How has e-monitoring created new roles in practice? What is the authority and legitimacy of these roles?
Systems thinking *Multidisciplinary*	Foster-Fishman et al. (56); Hawe et al. (57)	A system is set of objects whose interconnected linkage together forms a function or purpose. Theorizing the function and dynamics of ‘the whole' sheds light on the importance (or otherwise) of ‘the parts'.	Provides various schema to theorize the context/setting into which the electronic monitoring practice is introduced and appreciate how it might displace activity and align (or clash) with standards, normal, values, key functions, existing relationships, ongoing processes, history etc.	What resources and activities have become aligned with e-monitoring? What activities has e-monitoring displaced?
Complex adaptive systems thinking *Multidisciplinary*	Axelrod and Cohen (58)	Like above, but gives emphasis to the fact that the agents in the system (people, groups) have agency and take actions (adapt) to changes around them. From the multiple adaptations/actions comes emergent (new) properties of the system such as the capacity for the system to self-organize to achieve emerging new goals.	Using complexity science, the self-organizing properties of teams can be harnessed proactively in quality improvement interventions (59). The same observation has recently been made by May et al. (51) as an extension of Normalization Process Theory. This prompts consideration of whether introducing a new technology in a health promotion setting experiences and/or invites self-organization.	Has practice evolved new structures and routines? Is information for practice improvement being harnessed from new places?
Worldview theory *Anthropological theory*	Geertz (60); Rapport and Overing (61)	Examines a person or groups' picture of how things (e.g. oneself, society, the nature of things) actually are. Encompasses ideals, norms and values.	Known to influence the requirement to implement tobacco control policy in hospitals (62). Can also explain typologies of personal orientation to practice in community based health promotion (63).	How does e-monitoring fit with espoused and observed values of practice? What things are practitioners willing/not willing to give up as e-monitoring gets adopted?
Consolidated Framework for Implementation Research (CFIR) *Implementation framework*	Damschroder et al. (64)	Collates many different theories to explain the successful implementation of a new program or practice across 6 principal domains (44 constructs). These span different levels (e.g., individual, team/workplace setting, and policy environment).	Not designed to explain uptake of e-monitoring *per se*, but draws attention to similar factors of interest that may explain e-monitoring uptake e.g., the benefits or outcomes of the technology's use (acceptability of the health target to be achieved); self-efficacy of individuals; available resources; and external incentives. Because it derives from a systematic synthesis of extant research, the CFIR reflects the high volume of behavioral science research accumulated in implementation to date. It therefore may under-represent insights from social and political theory.	What organizational incentives and rewards support e-monitoring? How does an individual practitioner's sense of efficacy predict e-monitoring use?

^*^Sample questions are typical of the theory (or part thereof) but not necessarily exclusive to it.

On the more ecological side, Activity Settings Theory is about the dynamics of settings—spaces where people come together and carry out particular regularized actions (55). Activity Settings Theory invites an analysis of the act of e-monitoring in terms of whether it enriches, reconfigures, or strips the practice setting in terms of professional roles and resources (informational, relational, material, emotional, affirmational), or sets up time constraints or dynamics that enhance or impede other important functions of the practice system. It also invites interrogation of the visible symbols introduced into the setting by e-monitoring and whether they align or clash with the existing cultural norms. So, on the up side, does a person's ability to troubleshoot the software (a role) create new relationships? On the downside, do computers, software and graphic displays create workplace hierarchies that were not there previously? Do signs of officialdom start to crowd out the welcome messiness of everyday interaction? The theories tune researchers into what to look for and how it might matter. If there are not enough meaningful roles to be shared among the people in a setting, then alienation ensues. Alternatively, too many roles per person (meaningful or not) leads to exhaustion (65). Understanding these dynamics potentially leads to interventions that can be more effective and sustainable. So, e-monitoring could be crafted to create a dynamic that moves workplace wellbeing and effectiveness forward, through the use of some particular theory.

Collectively, these theories invite research that expands the questions asked about new technologies—beyond questions about whether technologies improve a particular health outcome—to issues that may be more important to the long-term strength and sustainability of the field of health promotion. That is, how are digital technologies intended to improve and support best practice?

## Concluding remarks

The lure of e-monitoring is that a practitioner can capture, store, analyse and communicate data in real time across geographical settings at the click of a button. The advantages of such systems, however, must be weighed against potential disadvantages. The onus turns to researchers in partnership with practitioners to design innovative studies to fully illuminate the experience of e-monitoring of health promotion practice and the full insights of what is being learned. A researcher-policy maker-practitioner partnership is currently undertaking an ethnography of an e-monitoring technology being used to track childhood obesity prevention programs in New South Wales, Australia (66). Likewise, future studies might usefully locate themselves within particular theoretical perspectives so that knowledge and understanding can be more easily identified, interpreted, extended and/or revised. This is critical if insight from research on e-monitoring from one context is to be used in another. For example, if an innovation is theorized to be purely technical and tested using a positivist orientation only (e.g., does use of the technology lead to increased physical activity in schools?) then such research will not explain the immediate disuse of the technology once the research process is over [as was the experience of Bors et al. (6)]. Nor will such research provide insights to overcome social resistance to the use of the technology in another setting.

Indeed, by far the biggest threat (or opportunity) accompanying the increasing uptake of e-monitoring of implementation in health promotion is the imperative placed on us to articulate practice itself and how good practice will be defined, supported and recognized. The point of distinction is whether we conceptualize good practice as a context of discovery (i.e., improvement science), or simply a context of program or practice delivery (i.e., implementation science) (39, 40). In terms of e-monitoring, an implementation science perspective might encourage teams to adopt and use a particular system whereas an improvement science perspective might consider how to design or use such systems in ways that facilitate practitioners' agency to “re-invent” programs and processes for local use (67). This idea of practice as re-invention aligns with May's assertion that practitioners “seek to make implementation processes and contexts plastic: for to do one thing may involve changing many other things” (50).

We therefore invite more improvement-science-oriented research to give shape to knowledge which reciprocally improves both e-monitoring and practice to foster inbuilt capability and innovation. We encourage developers of e-monitoring systems to share their learnings with the field, and to integrate programs of research into the roll out and implementation of e-monitoring systems. Finally, we join other colleagues in calling for future research to make clear the theoretical underpinning of research questions and approaches, and to consider a broad array of user perspectives into the impact and value of e-monitoring systems. Some time ago, health promotion researchers urged recognition that dissemination is a two-way process, insisting that knowledge from practice be given more consideration alongside getting knowledge into practice (68). Advancement of practice may not fully occur if e-monitoring acts to privilege one knowledge source more than the other. Fortunately, health promotion has never had a moment in history with better infrastructure to address this challenge, to represent what practice is, what practice can achieve, and how it can evolve meaningfully in the digital age.

## Author contributions

KC and PH co-conceptualized this manuscript. KC led the initial draft and PH co-wrote sections of the paper. Both contributed to the final drafting of the manuscript.

### Conflict of interest statement

The authors declare that the research was conducted in the absence of any commercial or financial relationships that could be construed as a potential conflict of interest.

## Footnotes

^1^This illustrative story is a composite narrative drawn from practice experience.
